# Effect of a single session of transcranial direct-current stimulation on balance and spatiotemporal gait variables in children with cerebral palsy: A randomized sham-controlled study

**DOI:** 10.1590/bjpt-rbf.2014.0053

**Published:** 2014

**Authors:** Luanda A. C. Grecco, Natália A. C. Duarte, Nelci Zanon, Manuela Galli, Felipe Fregni, Claudia S. Oliveira

**Affiliations:** 1 Programa de Pós-Graduação em Ciências da Reabilitação, Universidade Nove de Julho (UNINOVE), São Paulo, SP, Brazil; 2 Laboratory of Neuromodulation, Center of Clinical Research Learning, Spaulding Rehabilitation Hospital, Harvard Medical School, Boston, MA, United States; 3 Centro de Neurocirurgia Pediátrica (CENEPE), São Paulo, SP, Brazil; 4 Departamento de Neurocirurgia, Universidade Federal de São Paulo (UNIFESP), São Paulo, SP, Brazil; 5 Dipartimento di Bioingegneria, Politecnico di Milano, Milan, Italy

**Keywords:** cerebral palsy, physical therapy, movement, balance, electric stimulation, motor cortex

## Abstract

**Background:**

Transcranial direct-current stimulation (tDCS) has been widely studied with the aim of enhancing local synaptic efficacy and modulating the electrical activity of the cortex in patients with neurological disorders.

**Objective:**

The purpose of the present study was to determine the effect of a single session of tDCS regarding immediate changes in spatiotemporal gait and oscillations of the center of pressure (30 seconds) in children with cerebral palsy (CP).

**Method:**

A randomized controlled trial with a blinded evaluator was conducted involving 20 children with CP between six and ten years of age. Gait and balance were evaluated three times: Evaluation 1 (before the stimulation), Evaluation 2 (immediately after stimulation), and Evaluation 3 (20 minutes after the stimulation). The protocol consisted of a 20-minute session of tDCS applied to the primary motor cortex at an intensity of 1 mA. The participants were randomly allocated to two groups: experimental group - anodal stimulation of the primary motor cortex; and control group - placebo transcranial stimulation.

**Results:**

Significant reductions were found in the experimental group regarding oscillations during standing in the anteroposterior and mediolateral directions with eyes open and eyes closed in comparison with the control group (p<0.05). In the intra-group analysis, the experimental group exhibited significant improvements in gait velocity, cadence, and oscillation in the center of pressure during standing (p<0.05). No significant differences were found in the control group among the different evaluations.

**Conclusion:**

A single session of tDCS applied to the primary motor cortex promotes positive changes in static balance and gait velocity in children with cerebral palsy.

## Introduction

Transcranial direct-current stimulation (tDCS) is a widely studied innovative technique consisting of the application of low-intensity monophasic electrical current to the scalp. The electrical current flows from the electrodes and penetrates the skull, reaching the cerebral cortex. Although most of the current is dissipated among the overlying tissues, a sufficient amount of current reaches the structures of the cortex, modifying the membrane potential of the cells and modulating cortex activity^1^^,^ 2. It has been suggested that the effects of tDCS stem from persistent changes that resemble long-term potentiation and can lead to enhanced synaptic efficacy^3^.

There has been an increase in the number of studies stating that tDCS applied to the motor cortex can be used for the treatment of neurological disorders in children, such as cerebral palsy (CP)^4^. CP results in diminished activation of the central nervous system during the execution of movements^5^. A reduction in motor cortex excitability in children is associated with poor motor development^6^. Neurophysiological analyses have revealed global alterations in cortex excitability in children with CP, with a reduction in the activation of corticospinal and somatosensory circuits^7^. The reduction in somatosensory activation may be the neurological basis for poor tactile, proprioceptive and kinesthetic awareness in children with CP^8^. While there is no cure for the brain lesion associated with this condition, sequelae can be minimized through neurorehabilitation methods^9^. Studies involving functional magnetic resonance in children with CP have demonstrated that rehabilitation resources are capable of promoting the activation of the primary motor cortex^9^, which is an important area of the brain capable of facilitating cerebral reorganization^10^.

Ninety percent of children with CP exhibit impaired gait due to diminished cortex excitability, excessive muscle weakness, abnormal joint kinematics, and diminished postural reactions^11^. Moreover, inadequate postural control limits motor development in these children^12^^,^ 13.

Kaski et al.^14^ found that anodal tDCS induces changes in the excitability of the motor cortex referring to the lower limbs, with improvements in both balance and gait. The hypothesis of the present study is that a single session of anodal tDCS applied to the primary motor cortex in children with CP can momentarily potentiate motor patterns through the enhancement of cortex excitability and activation of corticospinal circuits. The authors believe that the facilitation of cortical excitability of the primary motor cortex may enhance motor control and velocity of motor responses in children with CP. In CP, deficits in spatiotemporal gait parameters and postural stability are notorious and generate a functional impairment of the child. Additionally the evaluation of the static balance and gait analysis are consecrated and scientifically valid techniques. For these reasons, the stabilometry and analysis of spatio-temporal parameters of gait were selected as outcomes of this study. The expected outcomes are an increase in gait velocity and reductions in the oscillation of the center of pressure (CoP) during standing in the anteroposteior and mediolateral directions. However, the changes would likely be lost after a few minutes due to the limitation of tDCS to a single session.

The aim of the present study was to determine the effect of a single session of tDCS applied to the primary motor cortex regarding immediate changes in spatiotemporal gait and oscillations of the CoP during standing in children with CP classified at levels I to III of the Gross Motor Function Classification System (GMFCS).

## Method

**Figure 1:**
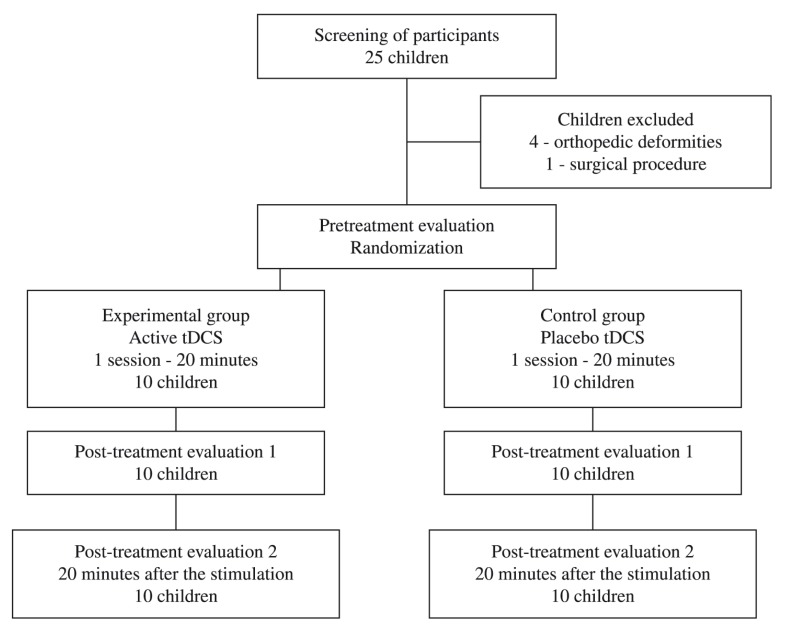
Flow diagram of study based on the Consolidated Standards of Reporting Trials (CONSORT) Statement.

The present randomized, sham-controlled, cross-sectional study (Figure 1) was carried out in compliance with the ethical standards of the Declaration of Helsinki and received approval from the Human Research Ethics Committee of *Universidade Nove de Julho* (UNINOVE), São Paulo, SP, Brazil, under process number 69803/2012. This study is registered with the Brazilian Registry of Clinical Trials (process RBR-9B5DH7). All guardians signed a statement of informed consent agreeing to the participation of their children.

Children with a diagnosis of spastic CP were recruited from specialized clinics. The inclusion criteria were classification at levels I, II and III of the GMFCS^15^^,^ 16, independent gait for at least 12 months, age six to ten years, and degree of understanding compatible with the procedures proposed. The following were the exclusion criteria: having undergone any surgical procedure or neurolytic block in the previous 12 months, orthopedic deformity, epilepsy, metal implants in the skull or hearing aids. Following the application of the eligibility criteria, 20 children were selected for the study.

The participants were randomized into the experimental and control groups based on the order of inclusion into the study. A randomization list was generated using blocks of six (for every six participants, three were randomly allocated to each group) and four (for every four participants, two were randomly allocated to each group) to minimize the risk of imbalance in the size of the groups.

The procedures were carried out in a single day. Following Evaluation 1 (pretreatment evaluation/ before stimulation), the children received 20 minutes of either active (experimental group) or sham (control group) tDCS. The children received tDCS at rest and seated comfortably. A responsible therapist accompanied the stimulation session. Evaluation 2 (post-treatment evaluation/after stimulation) was performed immediately following tDCS and Evaluation 3 (twenty minutes after stimulation) was performed after 20 minutes of rest. Three researchers carried out the procedures - two performed the evaluations and one performed the tDCS. The evaluations and tDCS were carried out in separate rooms to ensure the blinding of the examiners. Only the researcher in charge of the application of the tDCS was aware of the allocation of the children to the experimental and control groups.

### Transcranial direct-current stimulation

tDCS is the application of a low-intensity direct current on the scalp using two electrodes (anode and cathode). A sufficient amount of current penetrates the overlying tissues and reaches the structures of the motor cortex, modifying the neuronal membrane potential. Anodal stimulation enhances cortex excitability. The tDCS device (Soterix Medical Inc., USA) included two non-metallic sponge surface electrodes measuring 5 × 5 cm^2^ and moistened with saline solution. The children in the experimental group received anodal stimulation of the primary motor cortex and those in the control group received placebo transcranial stimulation. The anode was positioned over the primary motor cortex of the dominant hemisphere following the 10-20 international system of electrode placement for electroencephalography^17^ and the cathode was positioned in the supra-orbital region contralateral to the anode. The current was applied to the primary motor cortex for 20 minutes, during which the children remained seated. The tDCS device has a button that allows the operator to control the intensity of the current. Stimulation was gradually increased until reaching 1 mA and gradually reduced in the final 10 seconds. For sham stimulation, the electrodes were positioned in the same manner and the stimulator was switched on for 30 seconds. This procedure gave the children in the control group the initial sensation, but they did not receive electrical stimulation for the remainder of the session. This is considered a valid control procedure in studies involving tDCS^18^.

### Evaluation procedures

The evaluation of spatiotemporal gait variables (gait velocity, cadence, step length, stride length and step width) was performed using the SMART-D 140^(r)^ system (BTS Engineering, Italy) with eight infrared cameras, the SMART-D INTEGRATED WORKSTATION^(r)^ with 32 analog channels and a synchronized video system. After the determination of the anthropometric measures (height, mass, lower limb length, distance between the femoral condyles or diameter of the knee, distance between the malleolus or diameter of the ankle, distance between the anterior iliac spines, and thickness of the pelvis), passive markers were placed at specific reference points directly on the skin for the evaluation of each segment of the body. The markers were placed over C7 and the sacrum as well as bilaterally over the acromion, anterosuperior iliac spine, greater trochanter, femoral epicondyle, femoral wand, tibial head, tibial wand, lateral malleolus, lateral aspect of the foot at the fifth metatarsal head and at the heel (only for static offset measurements), as described by Davis et al.^19^. The Davis marker-set was chosen as the protocol of choice to acquire the movement of lower limbs and trunk based on Ferrari et al.^20^. After the child was familiarized with the process, at least six trials were performed along a 5-meter catwalk at a pace self-selected by each child. Three consistent trials of each lower limb were considered for analysis. All readings were performed by the same experienced researcher to ensure the reliability of the data collection. In the present study, only spatiotemporal and kinematic gait variables were identified and computed. The following spatiotemporal parameters were analyzed:

- velocity (m/s): mean velocity of progression;
- cadence: number of steps in a time unit (steps/min);
- stride length (m): longitudinal distance between successive points of heel contact of the same foot;
- step length (m): longitudinal distance between the point of initial contact of one foot and the point of initial contact of the contralateral foot;
- step width (m): distance between the rear end of the right and left heel centerlines along the mediolateral axis;
- stance phase: % of gait cycle that begins with initial contact and ends at toe-off of the same limb.

Mean and standard deviation values of gait velocity, cadence, step length, stride length, and step width were used for the statistical analysis.

Static balance was evaluated with the use of a force plate (Kistler model 9286BA), which allows stabilometric analysis through readings of oscillations of the CoP. The acquisition frequency was 50 Hz, captured by four piezoelectric sensors positioned at the extremities of the platform, which measured 40 × 60 cm. The data were recorded and interpreted using the SWAY software program (BTS Engineering) integrated to and synchronized with the SMART-D 140^(r)^. The child was instructed to remain in a quiet standing position on the platform, barefoot, arms alongside the body, gaze fixed on a point marked at a distance of one meter at the height of the glabellum, with heels aligned and an unrestricted foot base. The children classified at level III of the GMFCS^15^^,^ 16 used their usual gait assistance device, which was positioned off the force plate. The platform used has dimensions (600X400X35mm) that do not require the child to make great postural adjustments to position the gait assistance device off the platform. The children were instructed to keep the assistance device off the platform. The positioning of the device should allow a comfortable posture. The exact location of the device was marked on the floor with a white ribbon. The positioning was used in the three Evaluations to allow same condition assessment and comparative analysis^21^.

Readings of displacement from the CoP on the X (anteroposterior) and Y (mediolateral) axes were performed under two conditions: eyes open and eyes closed. Three acquisitions of 30 seconds were obtained for each condition and the average of the acquisitions was used in the statistical analysis. The outputs of the force platform allowed us to compute the CoP time series in the anteroposterior direction and the mediolateral direction. The output of the platform was processed to compute quantitative parameters in the time domain. The anteroposterior and mediolateral coordinates of the CoP trajectory underwent post-acquisition filtering using a low-pass filter with a cut-off frequency of 10 Hz. In the analysis, we identified and computed the range of CoP displacement in the anteroposterior direction (RANGEAP index) and the mediolateral direction (RANGEML index), expressed in mm^21^.

### Statistical analysis

The Kolmogorov-Smirnov test was used to determine the adherence of the data to the Gaussian curve. Parametric distribution was demonstrated, the data were expressed as mean and standard deviation values. To verify the effect of transcranial stimulation (active and placebo) over the three Evaluations in each group, intragroup analysis was performed. Intergroup analysis was performed to verify a possible effect obtained by the experimental group (active stimulation). With these goals, two-way analysis of variance (ANOVA) was used with the Bonferroni post hoc test, considering the variables: anteroposterior oscillations (open and closed eyes), mediolateral oscillations (open and closed eyes), and spatiotemporal gait parameters (gait velocity, cadence, step length, stride length, and step width). The level of significance was set to 0.05. The data was tabulated and processed using Statistical Package for the Social Sciences (SPSS, v.19.0).

## Results

Twenty children with CP were randomly allocated to the experimental group (active tDCS applied to the primary motor cortex) or control group (sham tDCS). No statistically significant differences between groups were found regarding the baseline data (age, anthropometric data, gait velocity, cadence, and static balance). Table 1 displays the anthropometric characteristics and functional classification of the children studied. All children tolerated the stimulation without complaints. Adverse effects were uncommon (three children) and restricted to redness and tingling of the skin in the experimental group.

**Table 1:** Anthropometric characteristics and functional classification of the participants.

	Experimental group (n=10)	Control group (n=10)
Age (years)*	7.2 (1.8)	7.8 (1.5)
Body mass (Kg)*	26.3 (3.2)	27.1 (2.6)
Stature (cm)*	125.8 (7.2)	126.1 (8.2)
Body mass index (Kg^2^/m)*	16.8 (1.2)	17.1 (1.1)
GMFCS (I\ II\ III)**	(3\4\3)	(3\4\3)
Topography (hemiparesis\diparesis)**	(4\6)	(3\7)

GMFCS - Gross Motor Functional Classification System

* Data expressed as mean (standard deviation)

** numbers indicate frequency (n) of children in each group.

**Figure 2:**
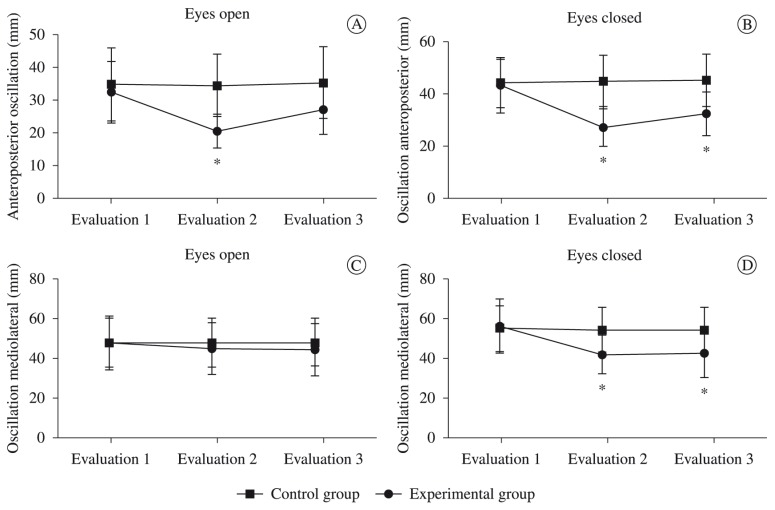
Results in the oscillations of the center of pressure before (Evaluation 1), immediately after (Evaluation 2), and twenty minutes after (Evaluation 3) the transcranial stimulation in the experimental group and the control group. A) Oscillation of the center of pressure in the anteroposterior direction with eyes open; B) Oscillation of the center of pressure in the anteroposterior direction with eyes closed; C) Oscillation of the center of pressure in the mediolateral direction with eyes open; D) Oscillation of the center of pressure in the mediolateral direction with eyes closed. Mean and standard deviation. *p<0.05.

Figure 2 is a description of the results obtained in the oscillations of the CoP. The experimental group showed a reduction in anteroposterior sway with eyes open in Evaluation 2 [F (2,36)=15.1, p=0.001], anteroposterior sway with eyes closed in Evaluations 2 [F (2,18)=29.3, p=0.001] and 3 [F (2,18)=17.8, p=0.001], and mediolateral sway with eyes closed in Evaluations 2 [F (2,18)=49.9, p=0.001] and 3 [F (2,18)=42.6, p=0.001]. The effects obtained also exhibited significant reductions in anteroposterior oscillation with eyes open (Pretreatment vs. Post-treatment 1 - effect: -11.8 mm, p<0.001; Pretreatment vs. Post-treatment 2 - effect: -5.2 mm, p=0.003), anteroposterior oscillation with eyes closed (Pretreatment vs. Post-treatment 1 - effect: -15.7 mm, p<0.001; Pretreatment vs. Post-treatment 2 - effect: -10.6 mm, p<0.001), mediolateral oscillation with eyes open (Pretreatment vs. Post-treatment 1 - effect: -2.7 mm, p<0.001; Pretreatment vs. Post-treatment 2 - effect: -3.1 mm, p<0.05), and mediolateral oscillation with eyes closed (Pretreatment vs. Post-treatment 1 - effect: -14.6 mm, p<0.001; Pretreatment vs. Post-treatment 2 - effect: -14.2 mm, p<0.001). In contrast, no significant differences among evaluations were found in the control group regarding gait variables or oscillations of the CoP. The control group showed no statistical difference in the intragroup analysis (p>0.05).

**Figure 3:**
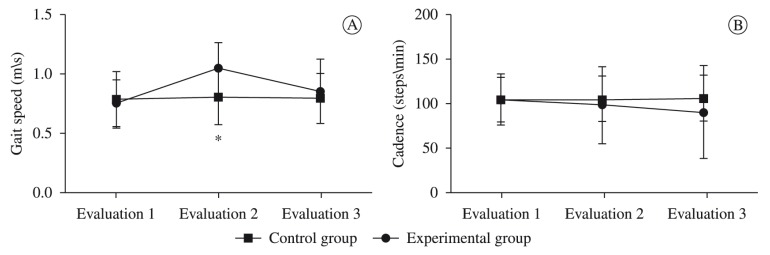
Results of gait velocity and cadence before (Evaluation 1), immediately after (Evaluation 2) and twenty minutes after (Evaluation 3) the transcranial stimulation in the experimental group and the control group. A) Gait velocity, B) Gait cadence. Mean and standard deviation. *p<0.05.

Table 2 describes the results obtained in the spatiotemporal gait variables. In experimental group, the statistical analysis showed an increase in walking speed in Evaluation 2 [F (2,18)=36.1, p=0.001], step length in Evaluation 2 [F (1,9)=19.3, p=0.017], and stride length in Evaluation 2 [F (2,36)=17.0, p=0.001] compared with the control group. No significant differences were identified in the control group (p>0.05). Figure 3 illustrates the results of gait speed and cadence.

**Table 2:** Performance at evaluation 1 (before stimulation), evaluation 2 (after stimulation), and evaluation 3 (twenty minutes after stimulation) of outcome of variables spatiotemporal gait.

	Experimental group			Control group		
	Evaluation 1	Evaluation 2	Evaluation 3	Evaluation 1	Evaluation 2	Evaluation 3
Gait velocity (m/s)	0.75 (0.19)	1.04 (0.21)	0.85 (0.27)	0.78 (0.23)	0.80 (0.20)	0.78 (0.21)
Cadence	104.6 (28.5)	98.3 (43.4)	90.6 (52.4)	103.5 (25.1)	105.3 (25.9)	104.2 (25.8)
Step length	0.33 (0.10)	0.40 (0.09)	0.34 (0.08)	0.35 (0.09)	0.34 (0.10)	0.34 (0.08)
Stride length	0.83 (0.01)	0.91 (0.07)	0.81 (0.06)	0.78 (0.10)	0.79 (0.07)	0.79 (0.10)
Step Width	0.15 (0.09)	0.16 (0.02)	0.15 (0.08)	0.16 (0.11)	0.16 (0.11)	0.16 (0.06)

## Discussion

tDCS currently occupies an important place in studies addressing neuromotor rehabilitation due to its potential in optimizing the results of physical therapy^14^^,^ 21 ^-^ 24. The authors of the present study were curious about the possible effects of tDCS performed in an isolated fashion regarding changes in postural stability and whether children would be able to tolerate the current. No previous studies were found addressing the effects of tDCS on postural control and gait in children with CP. Therefore, the aim of the present investigation was to determine the immediate effect of a single session of tDCS applied to the primary motor cortex in children classified at levels I to III of the GMFCS. To enhance the validity of the study, the experimental group (active tDCS) was compared to a control group (sham tDCS) and double-blind procedures (participants and examiners) were employed.

Three-dimensional gait analysis^25^^,^ 26 and stabilometry^26^^,^ 27 are considered fundamental assessment tools for the adequate quantification of the effects of interventions aimed at improvements in gait and static balance. These sensitive methods allow the identification of small changes within a short span of time and were therefore selected for the present study. The experimental group exhibited significant differences in the evaluations after the application of active tDCS regarding gait velocity and oscillations of the CoP in comparison with the evaluation held prior to stimulation.

The present study offers important findings. The experimental group exhibited an increase in gait velocity immediately following tDCS, but this increase was not maintained for more than 20 minutes after the end of the stimulation. Although this was a cross-sectional study involving a single session of tDCS, the results suggest that the momentary increase in cortex activation may have exerted an influence on motor control and gait. As the primary motor cortex was only stimulated for 20 minutes during rest, the authors did not expect the changes to be maintained in medium or long term. However, the findings could encourage future studies to combine tDCS with motor rehabilitation therapies to determine whether this technique can assist in improving gait and postural control in children with CP.

Gait velocity has an important relationship with the cadence. However, in this study there was an increase in walking velocity without increasing cadence. The authors believe that this fact can be explained by an increased step length.

Analyzing a population of elderly individuals (n=9) with leukoaraiosis, an ischemic lesion of the cerebral white matter that results in gait and balance disorders, Kaski et al.^24^ found that a single session of anodal stimulation in combination with gait and balance training had repercussions in the form of improvements in gait velocity, stride length, step length variability, and balance. In a study by Kaski et al.^14^, 30 healthy individuals received either active or sham tDCS to either the primary motor cortex or prefrontal cortex prior to walking on a moving platform (a mobile sled moved with a maximum velocity of 1.4 m/s). The group that received active tDCS exhibited an increase in gait velocity. Thus, anodal stimulation was capable of inducing changes in the excitability of the motor cortex of the lower limbs, thereby potentiating locomotion control. All of these previous findings underscore the potential of anodal stimulation of the motor cortex regarding the facilitation of motor recovery.

Balance deficit resulting in frequent falls is one of the most limiting aspects of CP^13^^,^ 26 ^,^ 27. Regarding oscillations of the CoP, two important findings were identified in the present study: 1) the similarity in the results with and without visual restriction; and 2) although a small number of participants were classified at level III of the GMFCS (three per group), the effects apparently involved these children, who require gait-assistance devices.

Visual compensation is an important aspect of postural stability. In children with CP, oscillations are greater with eyes closed due to the lack of visual compensation. The results suggest that there was a momentary improvement in postural stability. Although there are no studies that address the effects of tDCS on static balance, the authors believe that greater effectiveness of the proprioceptive system may have resulted from the stimulation of the cortical area. Thus, the motor responses were effective in minimizing the oscillations with visual restriction. Similar results on the effect size of the oscillations of the CoP, with and without visual restriction, are observed only with more dynamic interventions, such as the use of ankle-foot orthoses^26^.

All clinical effects observed following the application of tDCS are directly related to cortex modulations resulting from stimulation dependent on the polarity of the current. Anodal stimulation increases cortex excitability, favoring the depolarization of the neuronal membrane, whereas the cathode has an inhibitory effect through the hyperpolarization of the neuronal membrane^28^^,^ 29. A number of studies have demonstrated that tDCS is successful in achieving these effects, but some papers suggest that anodal stimulation applied to the primary motor cortex seems to have an effect that is dependent on the learning task and the formation of memory. These neurophysiological aspects and the clinical findings described in the results and discussion sections of this paper suggest that tDCS may be an important tool for potentiating the effects of neuromotor rehabilitation. Although the present investigation has limitations, such as not being a prospective study and not involving a broader stimulation protocol, important preliminary findings are described herein^30^. Such findings can offer a direction for the development of further studies that address the use of tDCS in combination with physical therapy to treat locomotion and postural disorders in children with CP.

## Conclusion

Based on the present findings, a single session of tDCS applied to the primary motor cortex in children with CP was capable of causing significant reduction in anteroposterior oscillation with eyes open and eyes closed and in mediolateral oscillation with eyes closed in comparison with the control group (tDCS sham). Moreover, increases in gait velocity, step length, and stride length were also observed after stimulation. However, the results were not maintained for more than 20 minutes after the end of stimulation.
